# Non-Destructive Detection of *Elasmopalpus lignosellus* Infestation in Fresh Asparagus Using VIS–NIR Hyperspectral Imaging and Machine Learning

**DOI:** 10.3390/foods15020355

**Published:** 2026-01-19

**Authors:** André Rodríguez-León, Jimy Oblitas, Jhonsson Luis Quevedo-Olaya, William Vera, Grimaldo Wilfredo Quispe-Santivañez, Rebeca Salvador-Reyes

**Affiliations:** 1 Facultad de Ciencias Agrarias, Universidad Nacional de Cajamarca, Av. Atahualpa N° 1050, Cajamarca 06002, Peru; arodriguezl@unc.edu.pe (A.R.-L.); or jimy.oblitas@upn.edu.pe (J.O.); jquevedol@unc.edu.pe (J.L.Q.-O.); 2 Facultad de Ingeniería, Universidad Privada del Norte, Vía de Evitamiento s/n cuadra 15, Cajamarca 06002, Peru; 3 Grupo de Investigación en Desarrollo e Innovación en Industrias Alimentarias (GIDIIA), Universidad Nacional de Frontera, Av. San Hilarión 101, Sullana 20103, Peru; wveraj@unf.edu.pe; 4 Escuela Profesional de Ingeniería Agroindustrial, Facultad de Ingeniería, Universidad Nacional Autónoma Altoandina de Tarma, Acobamba 120701, Peru; 5 Facultad de Ingeniería, Universidad Tecnológica del Perú, Lima 150101, Peru

**Keywords:** VIS–NIR spectroscopy, asparagus, feature selection, *Elasmopalpus lignosellus*, machine learning, SVM, hyperspectral imaging, damage detection, quality control

## Abstract

The early detection of internal damage caused by *Elasmopalpus lignosellus* in fresh asparagus constitutes a challenge for the agro-export industry due to the limited sensitivity of traditional visual inspection. This study evaluated the potential of VIS–NIR hyperspectral imaging (390–1036 nm) combined with machine-learning models to discriminate between infested (PB) and sound (SB) asparagus spears. A balanced dataset of 900 samples was acquired, and preprocessing was performed using Savitzky–Golay and SNV. Four classifiers (SVM, MLP, Elastic Net, and XGBoost) were compared. The optimized SVM model achieved the best results (CV Accuracy = 0.9889; AUC = 0.9997). The spectrum was reduced to 60 bands while LOBO and RFE were used to maintain high performance. In external validation (n = 3000), the model achieved an accuracy of 97.9% and an AUC of 0.9976. The results demonstrate the viability of implementing non-destructive systems based on VIS–NIR to improve the quality control of asparagus destined for export.

## 1. Introduction

Asparagus (*Asparagus officinalis*) is a perennial vegetable that is widely valued in the human diet due to its culinary versatility and nutritional profile, characterized by a high content of dietary fiber, vitamin C, polyphenols, and asparagusic acid [1,2]. This relevance supports large-scale agricultural activity, with global production reportedly exceeding 8.5 million tons in 2021. Production is primarily concentrated in China (86.4%), followed by Peru (4.3%), thereby consolidating both countries as key pillars of international supply [3]. Although more than 200 varieties have been documented, *A. officinalis* dominates the food market [4] and is commercialized mainly as a fresh product to meet consumer demand for unprocessed or minimally processed foods [2,5].

Fresh asparagus production faces a critical limitation due to tissue damage caused by the larva of *Elasmopalpus lignosellus*, which perforates internal structures and accelerates loss of turgidity, wilting, and stem deterioration [6]. Because damage is not always externally visible, infestation can compromise commercial quality, shorten postharvest life, and lead to rejections at packinghouses and export markets [7]. In supply chains, where appearance and firmness largely determine market value, late detection results in economic losses and increased sorting costs. Therefore, the industry requires rapid, reproducible, and non-destructive methods capable of identifying damage before it becomes visually evident, since manual inspection has limited sensitivity for early internal deterioration [8,9].

In Peru, the UC-157 F1 cultivar is particularly relevant from an economic standpoint [10,11]. However, routine quality control still relies mainly on visual inspection and manual handling, which are subjective approaches that depend on operator experience and show high variability between shifts [12,13]. Although the literature extensively addresses external defect detection using computer vision [14,15,16] and hyperspectral approaches [17], methods that reliably identify internal alterations associated with infestation (locally referred to as “picado”) under real operating conditions remain unclear. This technological shortfall directly affects competitiveness, increases waste, and undermines buyer confidence in export supply chains [18,19,20]. Improving detection capacity can reduce losses, strengthen compliance with standards, and contribute to the sustainability of the agro-export sector [19,20].

VIS–NIR hyperspectral imaging (HSI) has emerged as a non-destructive technology capable of capturing spatially resolved spectral information linked to physicochemical attributes such as water status, proteins, lipids, carbohydrates, pigments, and structural changes in plant tissues [21,22,23,24]. This capability enables the detection of subtle differences between damaged and healthy products even when deterioration is not visible to the naked eye [25,26]. Using machine learning, spectral patterns can be modeled to build robust classifiers that can discriminate complex and nonlinear signatures associated with biological damage [27]. Algorithms such as support vector machines (SVMs) and XGBoost have shown strong performance in capturing linear and nonlinear relationships in high-dimensional data [28,29], while multilayer perceptrons (MLPs) expand the capacity to represent spectral variability [30].

In crop diseases and pest damage, Zhang et al. [31] showed that an HSC-ResNet model, after selecting 76 key bands, achieved 95.51% accuracy when classifying five states, outperforming conventional ResNet. Ou et al. [31] combined 17 spectral bands and five vegetation indices in strawberry leaves inoculated with gray mold to train a CNN that achieved 96.6% accuracy and 96.3% recall. Dhakal et al. [32] classified wheat kernels damaged by Fusarium head blight using a boosting method with 97% accuracy, with a correlation to deoxynivalenol content of R^2^ = 0.75. Other studies have highlighted that wavelength optimization can maintain outstanding performance while enabling simpler and more deployable models. Dong et al. [33] identified 16 bands between 715 and 970 nm that yielded an F1-score of 99.8% when combined with random forest for distinguishing healthy citrus leaves from those with Huanglongbing symptoms. Yamada et al. [34] selected 31 bands in the 817–941 nm range and obtained accuracies from 80% to 100% for classifying cotton leaves according to the number of *Tetranychus urticae* mites, with 907.69 nm as the most discriminant wavelength. Collectively, these studies support the effectiveness of integrating feature selection and machine learning for high-precision phytosanitary monitoring; nevertheless, there are still no equivalent studies on internal larval infestation in asparagus spears.

Asparagus evaluation has also advanced through non-destructive technologies such as near-infrared (NIR) spectroscopy and hyperspectral imaging, which allow monitoring of quality, physiology, and health without altering product integrity. These approaches have been applied from postharvest control to early detection of stress and diseases, commonly supported by multivariate analysis and machine learning to interpret complex biological signals [35,36,37,38,39]. The integration of hyperspectral imaging with classification models, which has shown strong performance for identifying specific disorders, such as stem blight, through intelligent tissue discrimination, is a particularly promising development [40].

This technological evolution enables innovative solutions for the early identification of pest-related damage, thereby safeguarding the integrity of asparagus and supporting consistent quality assessment. By integrating hyperspectral images with classification models, an effective alternative emerges to automate the detection of “*picado*” (infestation), thereby reducing the variability associated with human judgment and enabling the standardization of sorting operations. This approach elucidates how infestation-driven physicochemical transformations modify VIS–NIR reflectance, providing quantitative evidence to refine evaluation protocols. From a technological standpoint, it also supports the exploration of reduced-band configurations that could be implemented in industrially deployed compact spectral sensors. Ultimately, the adoption of these tools may decrease postharvest losses, improve product uniformity, and enhance agroexport sector competitiveness. Therefore, this study aimed to evaluate the ability of VIS–NIR hyperspectral imaging and machine learning algorithms to predict *E. lignosellus* infestation in fresh asparagus spears.

Although hyperspectral imaging and machine learning have been widely used to detect diseases, stress, and external defects in horticultural products, their application to the detection of internal larval infestation in fresh asparagus spears remains limited. Damage caused by *E. lignosellus* develops mainly within the internal tissues and often shows no clear external symptoms at early stages, making conventional visual inspection particularly challenging [6,7]. In this context, the present study contributes to the application of established VIS–NIR hyperspectral imaging and machine learning techniques to a specific and underexplored phytosanitary problem under postharvest conditions. This work provides experimental evidence that internal infestation-related physiological and structural changes can be reliably captured through surface spectral responses by focusing on the basal region of the spear, where larval penetration typically occurs. These findings support the development of non-destructive inspection strategies that may enhance quality control, reduce postharvest losses, and improve the competitiveness of asparagus agroexport supply chains.

## 2. Methodology

### 2.1. Sample Acquisition

**Figure 1:**
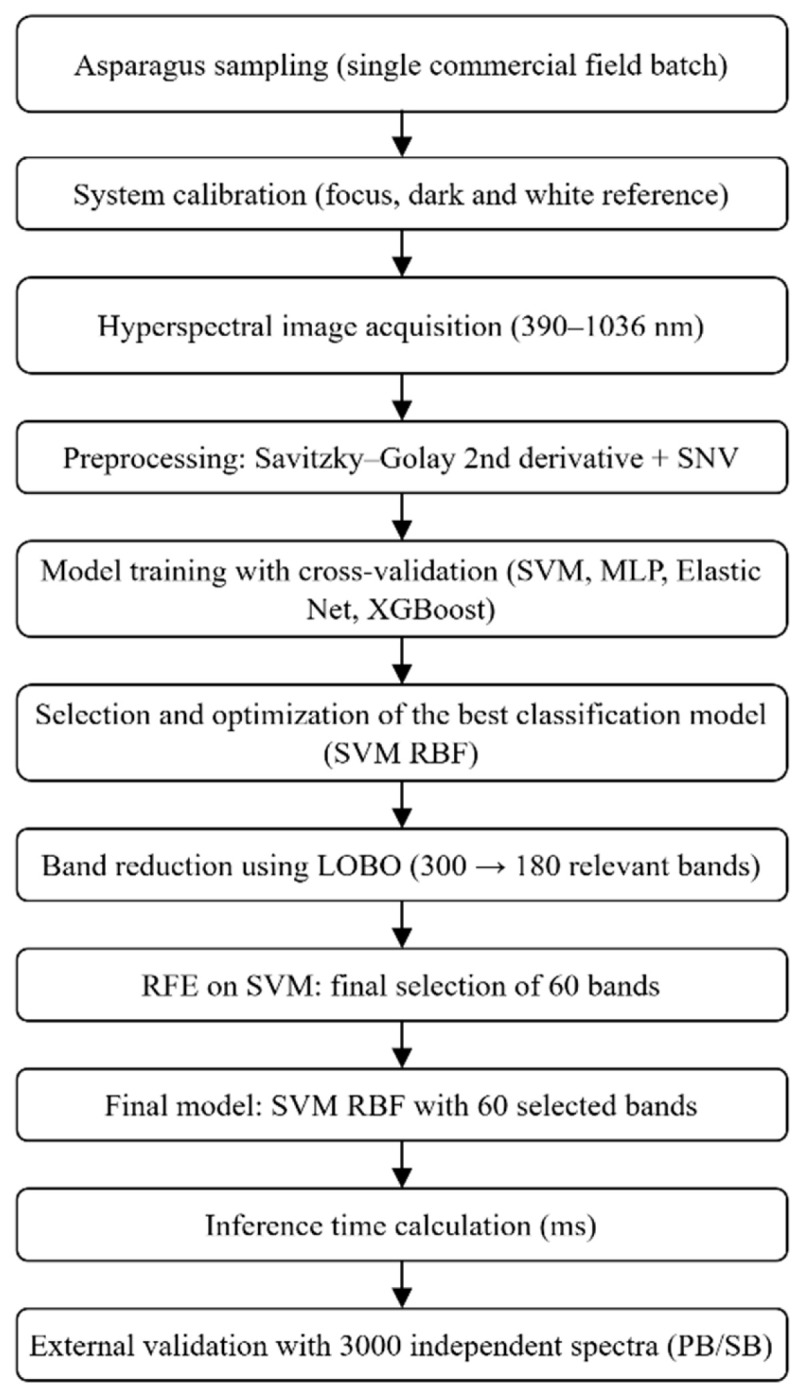
Diagram of the experimental development of the study.

A homogeneous batch of green asparagus (*Asparagus officinalis*), hybrid cultivar UC-157 F1, was harvested by a farmers’ association in La Libertad (Peru) under comparable field conditions and standardized postharvest handling (Figure 1). Upon reception, a phytosanitary assessment was performed by a plant health specialist, and spears presenting internal damage compatible with larval infestation by *E. lignosellus*, a boring pest associated with accelerated dehydration, wilting, and loss of firmness, were identified.

Following the initial non-destructive inspection, a destructive validation procedure was performed to confirm the presence and extent of internal infestation. Each preliminarily classified infested or healthy spear was longitudinally sectioned using a sterile blade, allowing direct visualization of the internal tissues. This procedure enabled the verification of the larval entry point, penetration depth, and degree of internal tissue alteration, which were used as the reference standard for final class assignment. Only spears with clear internal damage patterns consistent with *E. lignosellus* infestation were retained in the infested class, whereas those without visible internal alterations were assigned to the healthy class.

The phytosanitary assessment and destructive confirmation were conducted by a single plant health specialist with field experience in asparagus pest diagnosis to minimize labeling uncertainty. Although interobserver variability could not be quantified, the use of a single expert ensured intraobserver consistency throughout the labeling process. Moreover, the destructive validation step reduced subjectivity by providing direct anatomical evidence of infestation, thereby strengthening the ground truth reliability used for model training and evaluation.

For sampling, 10 kg of infested and 10 kg of sound asparagus were separated. From these lots, 900 spears with comparable morphology (length: 18–22 cm; diameter: 11–14 mm) were randomly selected to minimize potential bias due to size or developmental stage. Two balanced classes were defined: 450 infested spears (PB, “picado”) and 450 sound spears (SB). Class assignment was verified immediately before imaging to minimize labeling errors. All samples were maintained at controlled room temperature (18 °C) before imaging to limit the variability associated with postharvest dehydration.

**Figure 2:**
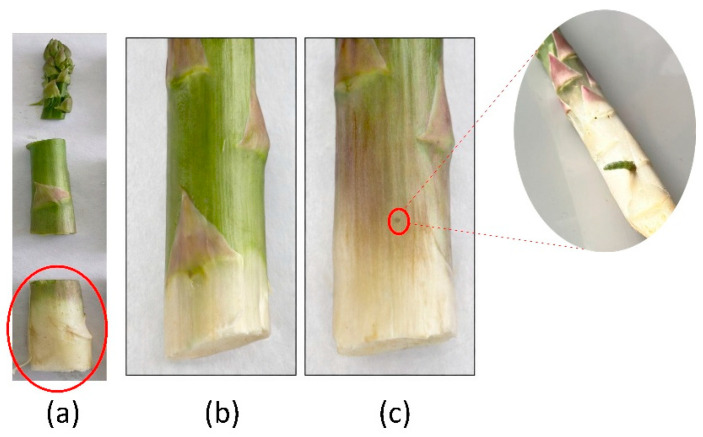
(**a**) Evaluation of the asparagus region (base), (**b**) sound stem (SB), and (**c**) infested stem (PB).

Prior to hyperspectral analysis, each asparagus stem was sectioned in a controlled manner so that HSI acquisition was performed exclusively on the base of the spear, the anatomical zone where larval perforation occurs. Supplementary Figure S1 provides a comparative assessment of classification performance across apical, middle, and basal sections, supporting the selection of the basal region for model development. Figure 2a shows the evaluated region, and Figure 2b,c show representative SB and PB examples, respectively. This procedure ensured a balanced dataset in which spectral differences could be primarily attributed to damage related to internal infestation.

### 2.2. Spectrum Acquisition and Preprocessing

Hyperspectral images were acquired by adapting the methodology applied by Vera et al. [41] using a Resonon Pika IR-Plus system (Resonon Inc., Bozeman, MT, USA) configured to operate in the spectral range of 390–1036 nm with constant spectral resolution.

The system was radiometrically calibrated prior to acquisition by adjusting the focus, acquiring a dark reference (to correct the dark current of the sensor), and collecting a white reference using a high-reflectance standard to normalize the signal and express the data as relative reflectance. After calibration, each asparagus sample was individually placed on the sampling plane and scanned under controlled illumination in pushbroom mode.

A region of interest (ROI) was defined from calibrated hyperspectral cubes of the basal portion, and a representative mean spectrum per sample was extracted by averaging all valid pixels within the ROI. Raw spectra were processed using a uniform preprocessing pipeline consisting of Savitzky–Golay second derivative of the second order (window length L = 27 points; polynomial order *p* = 2; derivative order m = 2), followed by standard normal variate normalization (SNV) to mitigate scattering effects, baseline shifts, and intensity differences associated with sample geometry.

A second-order derivative with a quadratic polynomial provides an effective balance between noise reduction and preservation of chemically relevant spectral features (Supplementary Table S2), enhancing inflection points without overfitting [42,43].

Mathematically, let $x_{i}\left(\lambda \right)$ be the spectrum of sample i at wavelength λ, sampled at equidistant points $\lambda_{j}$. The Savitzky–Golay filter locally estimates a polynomial of degree p within a window of L points (odd) and evaluates the derivative of order m. The local fit for a window centered at *j* is defined as

$$x_{i}\left(\lambda_{j+t}\right)\approx \sum_{k=0}^{p}a_{k}t^{k},\;t=-\frac{L-1}{2},\;\ldots ,\;\frac{L-1}{2}$$

The estimation of the derivative of order m at the center of the window is given as follows:

$$x_{i,SG}^{\left(m\right)}\left(\lambda_{j}\right)=\frac{m!}{{\left(\Delta \lambda \right)}^{m}}\sum_{t=-\frac{L-1}{2}}^{\frac{L-1}{2}}c_{t}^{\left(m\right)}x_{i}\left(\lambda_{j+t}\right)$$

where *L* = 27 (window length), *p* = 2 (polynomial order), *m* = 2 (derivative order), Δλ is the spectral step (spacing between wavelengths), $c_{t}^{\left(m\right)}$ are the coefficients of the SG filter for the derivative m, determined by least squares. Specifically, for the second derivative:

$$x_{i,SG}^{\left(2\right)}\left(\lambda_{j}\right)=\frac{2}{{\left(\Delta \lambda \right)}^{2}}\sum_{t=-13}^{13}c_{t}^{\left(2\right)}x_{i}\left(\lambda_{j+t}\right)$$

Following the derivative computation, SNV normalization was applied per sample:

$$x_{i,SNV}\left(\lambda_{j}\right)=\frac{x_{i,SG}^{\left(2\right)}\left(\lambda_{j}\right)-\mu_{i}}{\sigma_{i}}$$

where the mean $\mu_{i}$ and standard deviation $\sigma_{i}$ of the spectrum are calculated as follows:

$$\mu_{i}=\frac{1}{P}\sum_{j=1}^{P}x_{i,SG}^{\left(2\right)}\left(\lambda_{j}\right)\;\;\mathrm{and}\;\;\sigma_{i}=\sqrt{\frac{1}{P}\sum_{j=1}^{P}{\left(x_{i,SG}^{\left(2\right)}\left(\lambda_{j}\right)-\mu_{i}\right)}^{2}}$$

where *P* = 300 is the number of spectral bands. The preprocessing pipeline is summarized as follows:

$$x_{i,prep}=\mathrm{SNV}\left({\mathrm{SG}}_{L=27,p=2}^{\left(2\right)}\left(x_{i}\right)\right)$$

A final matrix of 900 samples × 300 spectral bands was obtained and used for model training and evaluation after preprocessing (Supplementary Data). Figure 1 summarizes the overall methodological workflow.

### 2.3. Model Construction and Validation

Supervised classification models widely used in spectroscopic applications were implemented: Support Vector Machine (SVM), multilayer perceptron (MLP), elastic net logistic regression, and XGBoost. The models were trained and evaluated using stratified five-fold cross-validation, preserving the PB/SB ratio within each fold.

The SVM used an RBF kernel, with regularization C ≈ 9 and **γ** = scale, enabling probability estimation for ROC-AUC calculation. Elastic Net was implemented as a logistic regression with elastic-net penalty, saga optimizer, and mixing parameter l1_ratio = 0.5, with regularization control via C. XGBoost was parameterized with regularization (reg_lambda, reg_alpha) with n_estimators = 500, learning_rate = 0.05, max_depth = 5, sampling subsample = 0.9, and colsample_bytree = 0.8. The MLP employed ReLU activation, Adam optimizer, and alpha = 1 × 10^−4^.

A comprehensive hyperparameter search was conducted using GridSearchCV to optimize the performance of the SVM, evaluating the combinations of the regularization parameter C (0.1–100) and kernel width γ (scale, 0.01–1.0). Optimization was performed within a pipeline integrating standardization and classification under stratified five-fold cross-validation, ensuring that scaling and model tuning were performed exclusively on training folds without accessing test data.

### 2.4. Dimensionality Reduction and Band Selection

Spectral optimization was conducted sequentially. First, LOBO analysis was carried out by grouping the spectrum into contiguous blocks of 30 bands along the wavelength axis. Each block was iteratively excluded, and the impact on classification performance was quantified to identify the most discriminating spectral regions. Based on LOBO, a reduced subset of 180 bands was retained.

Next, Recursive Feature Elimination (RFE) was applied to the reduced subset using a linear classifier (LinearSVC) as the base estimator to obtain an importance because its explicit weight vector enables direct band importance ranking, whereas nonlinear kernels (e.g., RBF) lack interpretable feature weights, limiting reliable feature elimination [44]. The target was a compact set of approximately 60 bands (Supplementary Material, Table S1) while preserving the full model’s discriminative information. Each reduction stage (300 → 180 → ~60) was evaluated using cross-validation to verify that spectral compression did not materially compromise predictive performance.

Importantly, both LOBO and RFE were applied as global dimensionality-reduction steps before cross-validation to simulate spectral optimization for practical deployment. The resulting cross-validation metrics should be interpreted as exploratory and potentially optimistic. Therefore, the independent external validation performed on a separate batch provides the primary and more reliable assessment of model generalization, which serves as the main evidence of predictive performance under realistic operating conditions.

### 2.5. Inference Time

The inference time was estimated through the repeated measurements (n = 100) of the prediction process using the trained SVM models. The time required to generate predictions for the evaluation set was recorded for each repetition. Computational performance of the full-spectrum model (300 bands) and the reduced model (~60 bands) was compared and expressed in milliseconds to quantify the effect of band reduction on runtime efficiency.

### 2.6. External Validation

Generalization ability was assessed through external validation using 3000 independent spectra obtained from later asparagus batches processed under the same acquisition and preprocessing procedure. This external dataset enabled the evaluation of model robustness against natural variability across batches and postharvest conditions not represented in the training set. The accuracy and ROC–AUC were used to report the performance, consistent with the internal validation scheme.

## 3. Results

### 3.1. Dataset Description

The dataset comprised 900 asparagus spears obtained from the same harvest batch to minimize variability associated with agronomic and postharvest differences. Half of the samples corresponded to SB and the other half to spears affected by *E. lignosellus* (“picado,” PB), whose larvae perforate internal tissues and may promote loss of turgidity, reduced water status, pigment alterations, and progressive microstructural changes.

**Figure 3:**
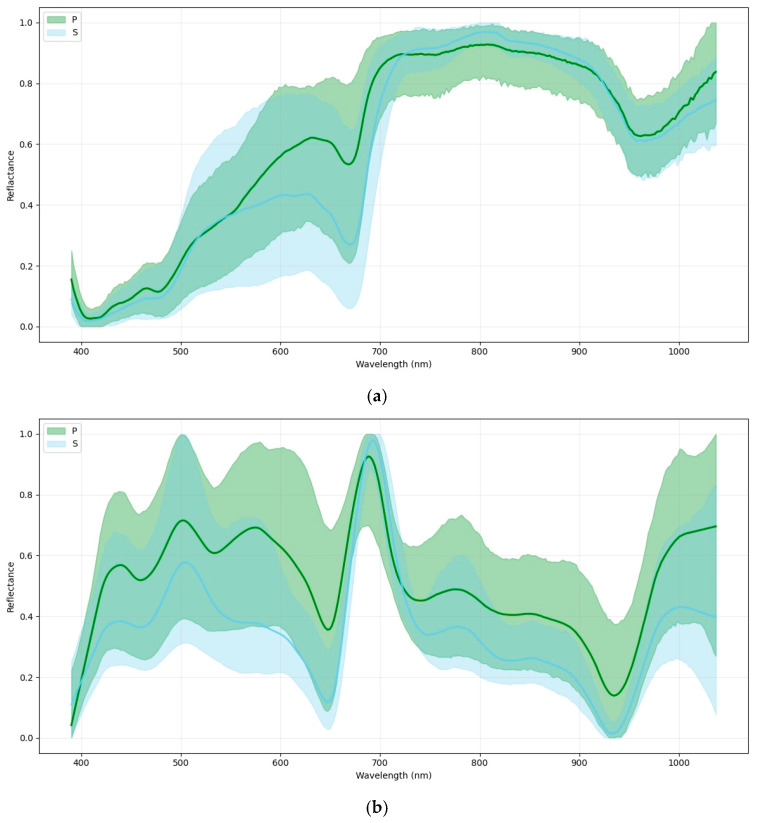
(**a**) Mean raw VIS–NIR reflectance spectra (390–1037 nm) of infested (PB) and sound (SB) asparagus. (**b**) Preprocessed spectra after Savitzky–Golay second derivative analysis and SNV normalization.

The average raw spectra (390–1036 nm) extracted from the basal portion showed consistent differences between classes (Figure 3a). In the visible range (≈400–700 nm), the PB spears exhibited lower reflectance, which is consistent with pigment-related changes. In the near-infrared region (≈700–1036 nm), differences were associated with tissue microstructure and water-related absorptions, particularly around the ~970 nm region (O–H overtones). After preprocessing using Savitzky–Golay second-derivative filtering and SNV normalization (Figure 3b), class-related differences became more evident by reducing scattering effects and baseline/intensity variability.

### 3.2. Model Training and Comparison

The preprocessed spectral matrix (900 samples × 300 bands) was used to train four classifiers: SVM (RBF kernel), MLP, Elastic Net, and XGBoost. All models were evaluated using stratified five-fold cross-validation.

Overall performance was high across the models; however, there were clear differences in both the central tendency and fold-to-fold variability. SVM achieved the highest mean accuracy (0.9889 ± 0.0062) and F1-weighted score (0.9889 ± 0.0062), as well as the highest ROC–AUC (0.9997 ± 0.0003). MLP yielded comparable performance (accuracy = 0.9850 ± 0.0056), whereas XGBoost and Elastic Net showed lower mean scores and larger dispersion across folds. The Elastic Net displayed the most pronounced decrease, which is consistent with the limitations of linear decision boundaries when modeling complex spectral interactions (Table 1).

**Figure 4:**
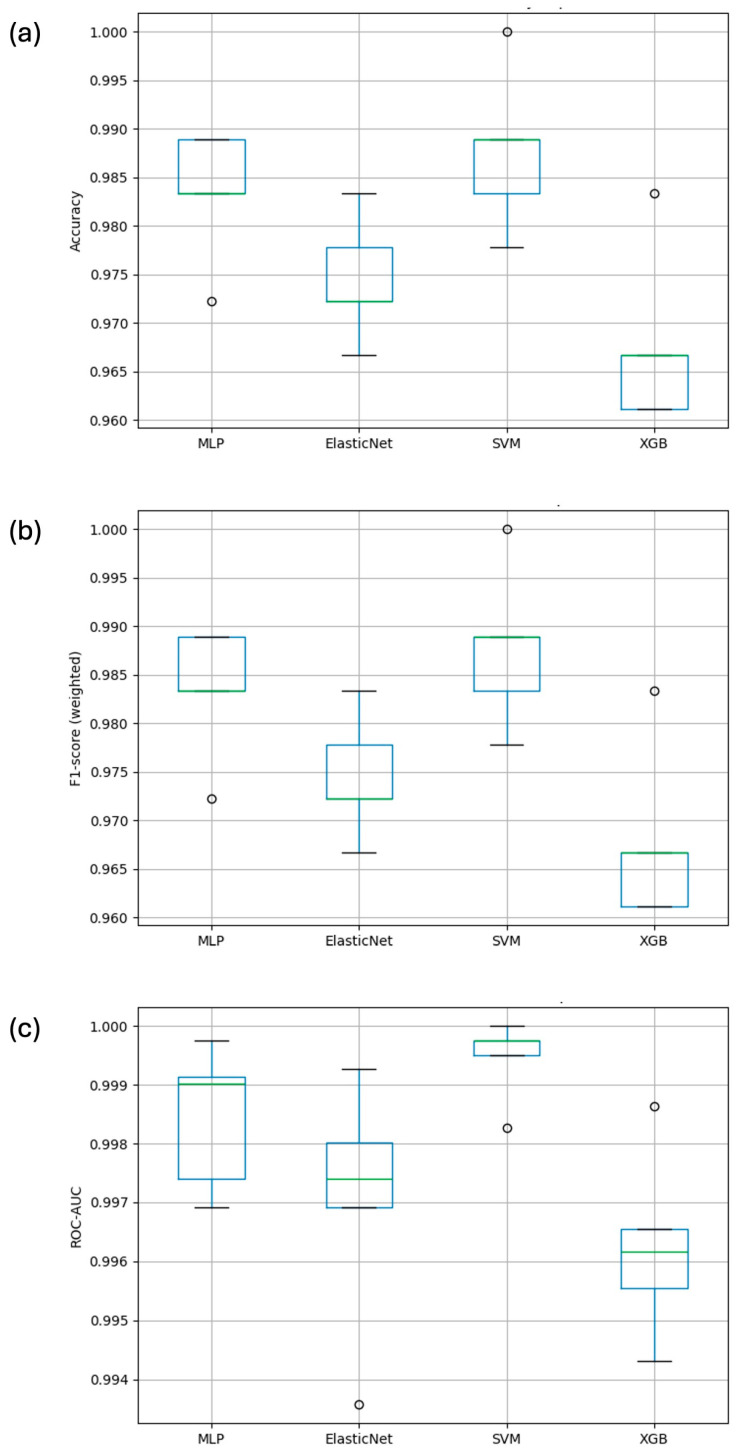
(**a**–**c**) Boxplots of each model’s performance measurement metrics.

The representation via boxplots (Figure 4) revealed that the SVM presented the lowest dispersion across all metrics, indicating greater robustness against physiological and instrumental variability, as well as subtle differences between stems. Figure 4a shows that the SVM maintained the highest median accuracy with minimal variation, while Figure 4b demonstrates consistent F1-score performance across validation folds. Furthermore, the ROC-AUC boxplot (Figure 4c) confirmed the SVM’s superior stability compared with other models. This stability is a key attribute in industrial applications where reproducibility is required under real operating conditions.

### 3.3. Optimization of the Best Model

Given its superior cross-validation performance, the SVM classifier was further optimized using GridSearchCV by evaluating 36 hyperparameter combinations (180 runs). The best-performing configuration corresponded to C = 10 and γ = “scale”. Under this configuration, cross-validation yielded accuracy = 0.9878 ± 0.0074, F1-weighted = 0.9878 ± 0.0074, and ROC–AUC = 0.9995 ± 0.0006 (Table 2), which is consistent with the strong performance of the initial model comparison.

**Figure 5:**
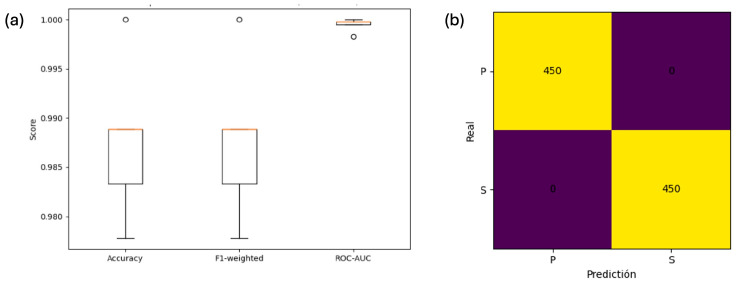
Evaluation metrics and analysis of the optimized SVM model. Note: Panel (**a**) presents boxplots of performance metrics obtained from stratified five-fold cross-validation, reflecting variability across folds. Panel (**b**) shows the confusion matrix derived from the final optimized SVM trained on the full dataset, provided for illustrative in-sample separability and not as a cross-validated estimate.

Figure 5a shows the distribution of cross-validation metrics for the optimized SVM, confirming consistently high performance across folds. Figure 5b presents the confusion matrix obtained by applying the optimized SVM to the complete dataset, illustrating the strong separability between the PB and SB classes under in-sample evaluation. Supplementary Figure S2 shows the averaged out-of-fold confusion matrix across the 5-fold cross-validation to provide a fold-independent estimate.

### 3.4. Strategic Band Reduction

Band reduction was performed to preserve predictive accuracy while improving interpretability and computational efficiency. This analysis was conducted using a global band-reduction step to simulate spectral optimization before the deployment of the model.

**Figure 6:**
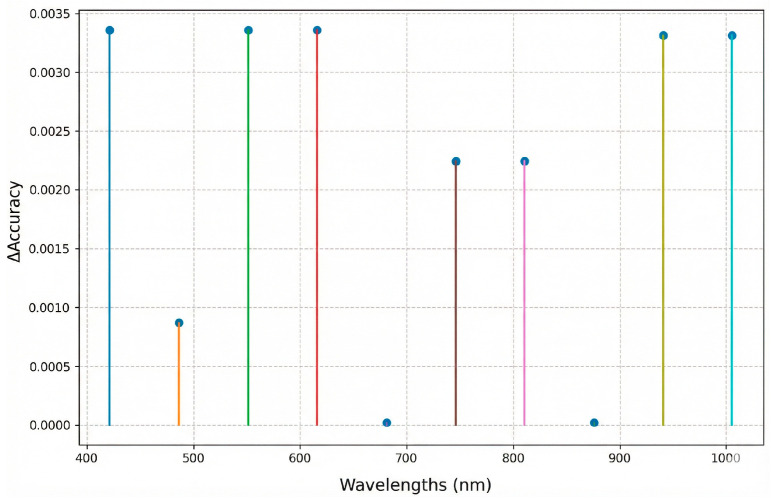
Sensitivity analysis of the optimized SVM model.

The sequential LOBO → RFE procedure evaluated the relative importance of spectral regions and individual wavelengths. LOBO identified relevant regions, yielding a reduced subset of 180 bands (60% of the original spectrum). Cross-validation performance using this subset was maintained and slightly improved within variability (accuracy = 0.9911 ± 0.0057; Figure 6), indicating that discrimination-relevant information is concentrated in specific spectral regions and that less informative bands can be removed to reduce noise. Because this analysis relies on a global band-reduction step, these cross-validation results should be interpreted as indicative of an optimist. Therefore, external validation provides primary evidence of model generalization.

**Figure 7:**
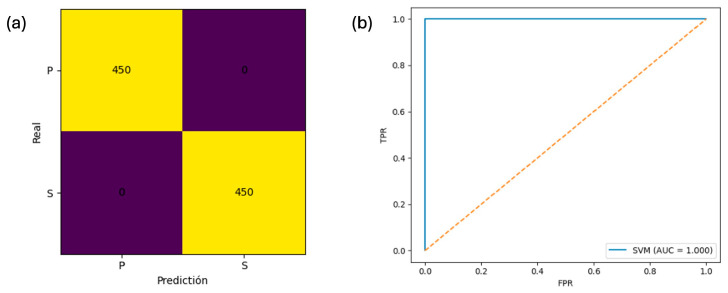
In-sample performance analysis of trained reduced SVM model. Note. Panel (**a**) present the confusion matrix derived from the final optimized SVM trained on the reduced dataset. Panel (**b**) shows the area under the curve.

Subsequently, RFE applied to the 180-band subset selected a compact set of 60 wavelengths (Table S1). The performance of the reduced SVM model was comparable to that of the full model (accuracy = 0.9900 ± 0.0074; F1-weighted = 0.9900 ± 0.0074; ROC–AUC = 0.9997 ± 0.0003; Figure 7), supporting the feasibility of deploying a reduced-band configuration without loss of discriminative capacity. Supplementary Figure S3 shows the ROC curves across cross-validation folds for the full and reduced models.

### 3.5. Comparison of Models by Inference Time

**Figure 8:**
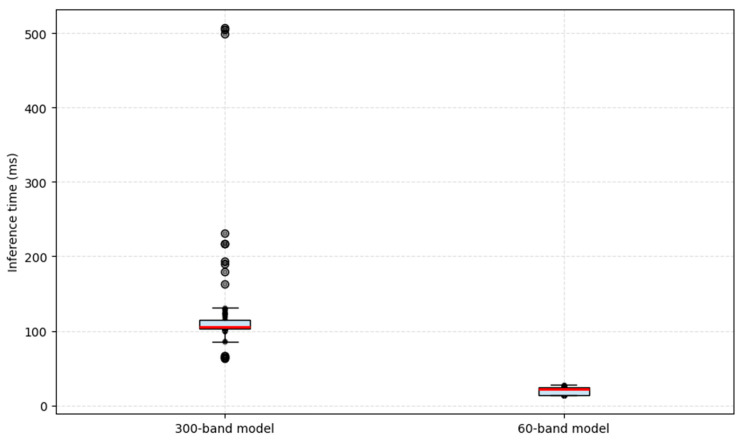
Comparison of the inference times between the full and reduced models.

Figure 8 compares the inference time between the SVM model trained with the full spectrum (300 bands) and the reduced model with 60 selected bands. The full model presented a mean inference time of 122.12 ± 48.76 ms, while the reduced model reached 39.20 ± 13.48 ms, indicating a reduction of 67.9% in prediction time. This decrease confirms that spectral reduction significantly improves computational efficiency without compromising classification performance, thereby strengthening the model’s viability for NRTI applications.

### 3.6. External Validation

**Figure 9:**
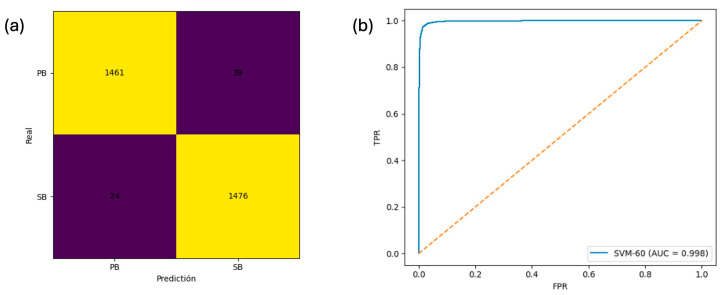
Performance of the final reduced SVM model on an external validation dataset. Note. Panel (**a**) present the confusion matrix derived from the external validation. Panel (**b**) shows the area under the curve.

The generalization performance was evaluated using an independent external dataset of 3000 asparagus spears (1500 PB, 1500 SB). The 60-band SVM achieved accuracy = 97.90%, F1-weighted = 0.9790, and a ROC–AUC = 0.9976 (Figure 9a,b), confirming its strong applicability under operational variability, although training was conducted on a single batch and broader multi-batch validation remains a future objective. The confusion matrix indicates that misclassifications were limited (PB→SB = 39; SB→PB = 24), which may reflect borderline physiological conditions where mildly infested spears present spectral signatures similar to sound spears, potentially compounded by natural dehydration variability across samples.

## 4. Discussion

In this study, infested asparagus exhibited a marked decrease in reflectance within the visible region (approximately 400–700 nm), consistent with pigment-related alterations and physiological stress associated with tissue damage. These spectral variations can be explained by larval infestation-induced physiological and structural changes [6]. *E. lignosellus* larvae perforate the stem at ground level, feed on internal tissues, and cause wilting, water transport disruption, tissue necrosis, and reduced turgidity. These processes accelerate moisture loss, compromise cell wall integrity, and affect the abundance and functionality of photosynthetic pigments such as chlorophyll, ultimately modifying how light interacts with the tissue surface.

The physical detection mechanism is an important consideration. Since visible light (400–700 nm) has limited penetration into plant tissue [45], the system does not directly detect larval perforation but instead captures physiological consequences manifesting in the outer stem layers. Larval activity disrupts water and nutrient transport, leading to localized dehydration, turgor loss, and cellular stress in cortical and epidermal tissues [6,46]. These changes alter the internal structure, water content, and pigments of the affected area [47,48,49], thereby modifying how light interacts with the surface of asparagus [50,51,52,53]. Despite the internal origin of damage, the spectrally measured surface signature reliably indicates the internal tissue condition, as confirmed by the strong correlation between spectral classification and destructive validation.

This detection principle, which is the surface manifestation of internal damage, aligns with observations in other crop pathogen systems. Ou et al. [31] reported decreased visible reflectance in strawberry leaves infected with gray mold due to pigment deterioration and water stress. However, important biological differences exist: asparagus damage is primarily internal within compact stem tissues, whereas strawberry alterations are superficial and foliar. These differences in tissue type, anatomical structure, and damage spatial localization explain variations in spectral magnitude and distribution despite similar directional trends in the visible region.

This study also demonstrated that an SVM with an RBF kernel outperformed Elastic Net and XGBoost in terms of discrimination performance (accuracy = 0.9889). This supports the existence of nonlinear relationships between reflectance patterns and damage caused by internal infestation. In line with the importance of nonlinear modeling for complex spectral–pathology patterns, Zhang et al. [54] reported that an HSC-ResNet model reached 95.51% accuracy when classifying quarantine diseases in apples, reinforcing the multidimensional nature of spectroscopy-based diagnosis. The contrast between studies suggests that, while deep learning can be highly effective, classical nonlinear classifiers, such as SVM, may provide excellent generalization when sample morphology and acquisition conditions are relatively controlled, allowing a soft-margin approach to capture discriminative structure without requiring deeper architectures.

Building on this performance, strategic band reduction offers practical advantages by supporting simplified sensing solutions and reducing computational cost. Reducing the input from 300 to 60 wavelengths maintained accuracy (0.9900), indicating that discrimination-relevant information is concentrated in specific spectral regions. Similarly, Dong et al. [33] achieved an F1-score of 99.8% for Huanglongbing detection using only 16 key bands. However, the requirement of a larger set (60 bands) in asparagus compared with 16 bands in citrus likely reflects the more subtle, heterogeneous, and internally distributed nature of larval damage, demanding broader spectral coverage to preserve physiologically meaningful information.

The high accuracy achieved with only 60 spectral bands and the short inference time suggest that the technique has potential for online implementation [55,56]. However, controlled and stable lighting conditions remain essential for reliable measurements [57,58]. The spectral reduction enables the transition from complex hyperspectral imagers to more affordable and faster multispectral cameras, thereby reducing costs and simplifying operation [59]. Future work should test the model under various growing conditions and develop a compact, user-friendly prototype for industrial deployment [60,61,62].

To assess real-world robustness, external validation was performed on 3000 independent samples. The 60-band model achieved an accuracy of 0.9790, indicating adequate generalization without evident overfitting. Dhakal et al. [32] reported an accuracy of 97% for wheat kernels affected by Fusarium head blight. Although both studies demonstrate robust generalization, the moderate decrease in asparagus performance may be partly explained by natural variability in water status and postharvest dehydration. Mildly infested and dehydrated yet healthy spears can exhibit partially overlapping spectral signatures in regions influenced by water absorption and tissue microstructure, increasing borderline cases and misclassification risk in operational settings.

Finally, the comparatively lower performance of the Elastic Net (accuracy = 0.9600) demonstrates that infestation-related spectral transformations do not follow simple linear patterns. Consistent with this finding, Yamada et al. [34] showed that nonlinear models, such as neural networks and random forest, substantially outperformed linear classifiers in detecting *T. urticae*. Pest-induced physiological phenomena, water loss, microstructural disorder, and optical alterations manifest through non-additive complex absorption and scattering behaviors where band interactions are non-additive. Thus, both studies underscore the need for nonlinear classifiers to accurately represent plant bio-optical responses and achieve robust diagnostic performance.

## 5. Conclusions

Spectral analysis showed that *E. lignosellus*-affected asparagus spears exhibit consistent optical differences in the visible and near-infrared regions. These differences are consistent with infestation-related physiological and structural changes, including pigment-associated variation and water status/microstructure alterations, supporting the ability of VIS–NIR hyperspectral signatures to discriminate between sound and damaged asparagus.

The best performance among the evaluated classifiers was achieved by the SVM model with an RBF kernel, with a mean cross-validation accuracy of 0.9889 and low fold-to-fold variability. This result indicates that effective discrimination relies on capturing nonlinear relationships between spectral features and damage caused by internal infestation. In addition, the sequential LOBO → RFE strategy reduced the input from 300 to 60 bands while maintaining high performance (accuracy = 0.9900), demonstrating that the most informative spectral content is concentrated in specific wavelength regions and that informed band selection can reduce computational complexity without compromising predictive capacity.

External validation on 3000 independent asparagus spears yielded an accuracy of 0.9790 and an area under the curve (AUC) of 0.9976, confirming that the reduced-band model generalizes beyond the training data. The observed stability suggests robustness to natural physiological variability across batches and a low risk of overfitting under the evaluated conditions. Conversely, the comparatively lower performance of the Elastic Net (accuracy = 0.9600) reinforces that linear models are unsuitable for representing the complex spectral patterns associated with heterogeneous biological damage.

Overall, these findings support the feasibility of VIS–NIR hyperspectral imaging as a rapid screening approach for detecting infestation-related damage in export asparagus, particularly when focusing on the basal portion of the spear. Future work should assess model stability under broader agronomic and postharvest conditions and further refine band-selection strategies with physical interpretability, enabling progress toward compact multispectral sensors or customized cameras for industrial deployment.

## Figures and Tables

**Table 1:** Cross-validation results (fivefold).

Model	Accuracy CV ± SD	F1-Weighted ± SD	ROC-AUC ± SD
SVM (RBF)	0.9889 ± 0.0062	0.9889 ± 0.0062	0.9997 ± 0.0003
MLP	0.9850 ± 0.0056	0.9850 ± 0.0056	0.9992 ± 0.0005
XGBoost	0.9660 ± 0.0150	0.9660 ± 0.0152	0.9965 ± 0.0010
ElasticNet	0.9600 ± 0.0200	0.9600 ± 0.0202	0.9940 ± 0.0018

**Table 2:** Cross-validation metrics of the optimized SVM model.

Metric	Result
Accuracy CV	0.9878 ± 0.0074
F1-weighted CV	0.9878 ± 0.0074
ROC-AUC CV	0.9995 ± 0.0006

## Data Availability

The original contributions presented in this study are included in the article/Supplementary Materials. Further inquiries can be directed to the corresponding authors.

## Supplementary Materials

The following supporting information can be downloaded at: https://www.mdpi.com/article/10.3390/foods15020355/s1, Supplementary Figure S1: Performance by spear section (apical, middle, and base). Supplementary Figure S2: Out-of-fold confusion matrix (5-fold CV) of the optimized SVM. Supplementary Figure S3: Receiver operating characteristic curves across folds (full vs. reduced model). Supplementary Table S1: Selected wavelengths (60 bands) used in the reduced model. Supplementary Table S2: Performance by type of preprocessing. Supplementary data. Band selection and model evaluation available at: https://github.com/Andr3ide/Esparragus-data/tree/8e78693e62c442cf348f57a1efc28c3f45abac3c (accessed on 10 January 2026).

## Abbreviations

The following abbreviations are used in this manuscript:

Abbreviation	Definition
AUC	Area Under the Curve
CNN	Convolutional Neural Network
CV	Cross-Validation
HLB	Huanglongbing
HSI	Hyperspectral Imaging
LOBO	Leave-One-Block-Out
MLP	Multilayer Perceptron
NIR	Near-Infrared
PB	Infested asparagus (Picado)
RBF	Radial Basis Function
ReLU	Rectified Linear Unit
RFE	Recursive Feature Elimination
ROC	Receiver Operating Characteristic
ROI	Region of Interest
SB	Healthy asparagus (Sano)
SD	Standard Deviation
SNV	Standard Normal Variate
SVM	Support Vector Machine
VIS	Visible
VIS–NIR	Visible and Near-Infrared
