# Supplementary material for: Non-Destructive Detection of Elasmopalpus lignosellus Infestation in Fresh Asparagus Using VIS–NIR Hyperspectral Imaging and Machine Learning
Source: Foods. 2026 Jan 19;15(2):355. doi: 10.3390/foods15020355 (PMC12840969; doi:10.3390/foods15020355)
Supplement: Supplementary file 1 [file foods-15-00355-s001.zip › foods-4095092-supplementary.pdf]

Supplementary Figure S1. Performance by spear section (apical, middle, and base).

The asparagus base was selected for infestation detection because it achieved the highest accuracy (0.994) compared to the apical (0.950) and middle sections (0.961). Using the Support Vector Machine, the best-performing model, confusion matrix results consistently confirmed the base as the most discriminative section.

Spear Sectión	Accuracy	Confusion matrix
APICAL	0.950	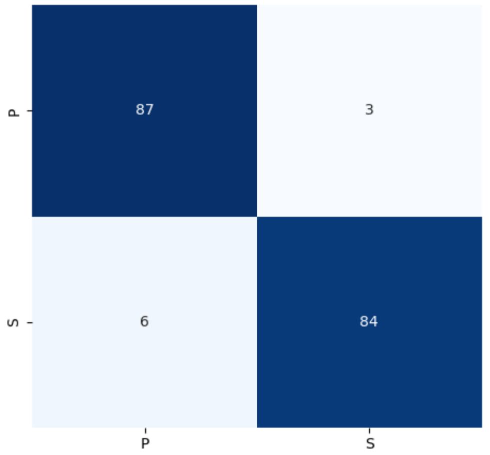
MIDDLE	0.961	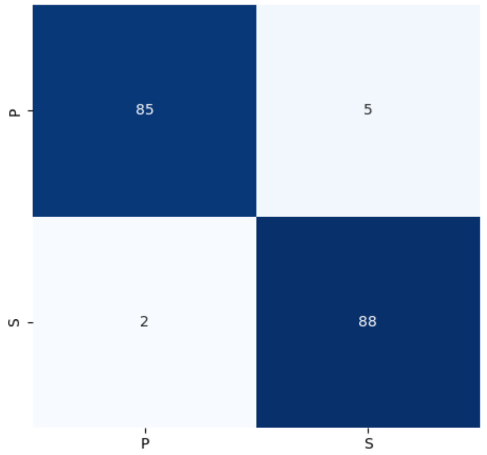
BASE	0.994	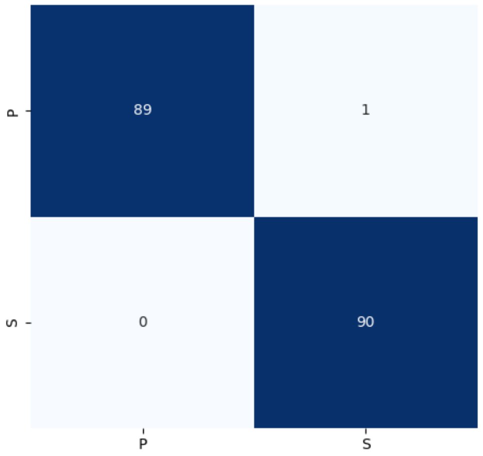

Supplementary Figure S2. Out-of-fold confusion matrix (5-fold CV) of the optimized SVM.

This averaged confusion matrix from 5-fold cross-validation shows very stable performance of the optimized SVM. Most samples are correctly classified (≈89 true negatives and ≈88.8 true positives), while misclassifications are minimal (≈1 false positive and ≈1.2 false negatives), indicating strong discrimination and low classification error across folds.

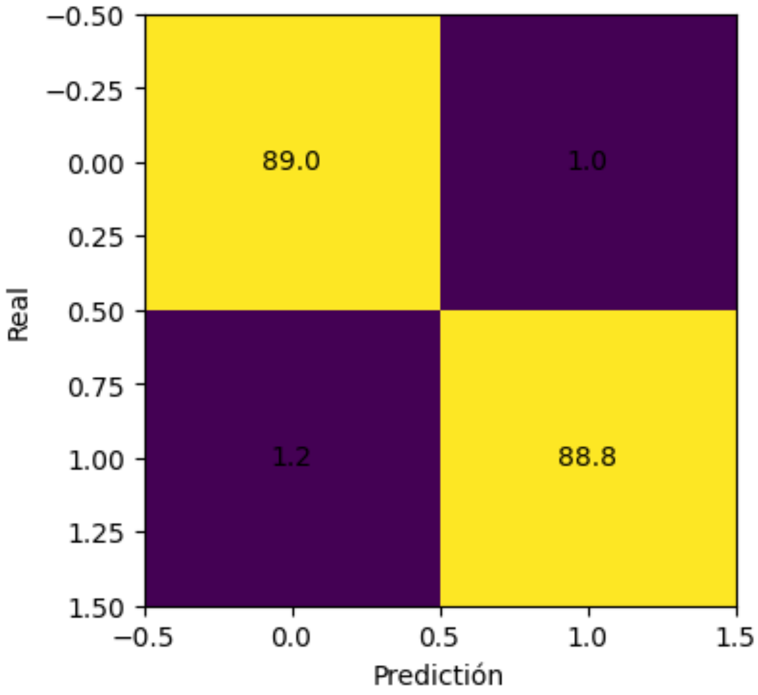

Supplementary Figure S3. Receiver operating characteristic curves across folds (full vs reduced model).

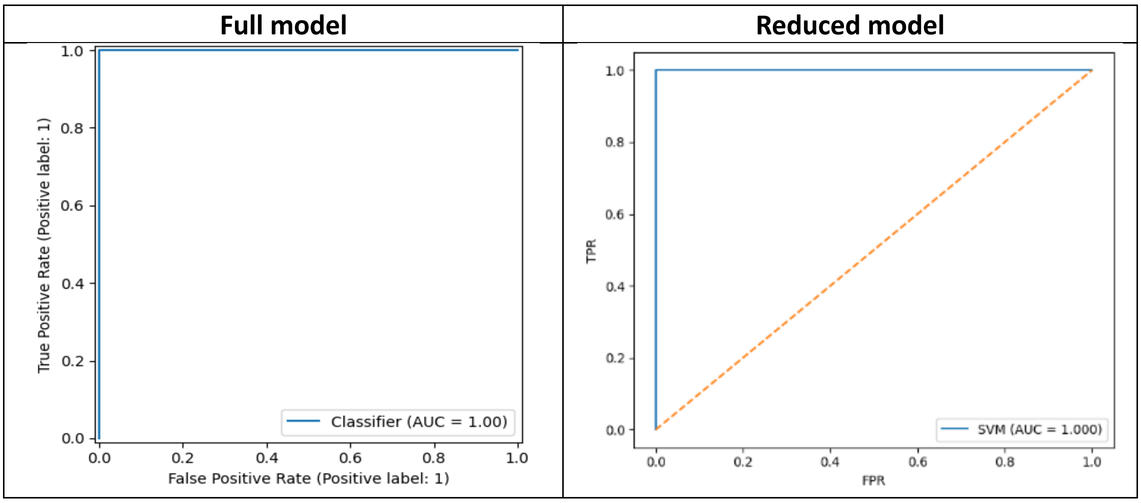

Supplementary Table S1. Selected wavelengths (60 bands) used in the reduced model.

N°	Wavelenghts
1	392.23
2	396.31
3	425
4	427.06
5	429.12
6	435.28
7	439.4
8	441.46
9	449.72
10	453.85
11	455.92
12	460.05
13	462.12
14	464.19
15	466.26
16	468.34
17	470.4
18	472.48
19	474.55
20	501.59
21	503.67
22	505.76
23	516.2
24	518.3
25	524.58
26	528.76
27	535.06
28	537.16
29	539.26
30	541.36
31	547.66
32	549.76
33	572.96
34	575.07
35	579.3
36	583.53
37	585.65
38	592.01
39	594.13
40	596.25
41	626.04
42	628.17

43	634.58
44	638.86
45	705.53
46	714.19
47	740.26
48	748.96
49	751.14
50	753.32
51	757.7
52	773
53	775.18
54	781.76
55	792.73
56	801.52
57	803.72
58	830.18
59	832.4
60	834.61

Supplementary Table S2. Performance by type of preprocessing.

In this study, Savitzky–Golay preprocessing was implemented using the second derivative (m = 2) with a second-order polynomial (p = 2), a configuration supported by spectroscopic evidence as a practical balance between noise suppression and preservation of chemically meaningful features. The second derivative enhances subtle inflection points and improves separation of overlapping bands, while a quadratic polynomial captures local curvature without introducing undue distortion or overfitting (Wilkins et al., 2023; Keshavarz et al., 2025). Notably, Supplementary Table S2 shows that SVM performance remained essentially unchanged across the tested settings (m = 1, p = 2; m = 2, p = 2; m = 2, p = 3), indicating that classification was robust to these preprocessing choices; therefore, m = 2, p = 2 was retained as a parsimonious and well-supported option for the subsequent analyses.

**Type of preprocessing**	**SVM accuracy**
Derv = 1<br>Poly = 2	Accuracy : 0.994<br>F1-score : 0.994<br>Matriz de confusión:<br>[[89 1]<br>[0 90]]
Derv = 2<br>Poly = 2	Accuracy : 0.994<br>F1-score : 0.994<br>Matriz de confusión:<br>[[89 1]<br>[0 90]]
Derv = 2<br>Poly = 3	Accuracy : 0.994<br>F1-score : 0.994<br>Matriz de confusión:<br>[[89 1]<br>[0 90]]
